# Two closely spaced missense *COL3A1* variants in *cis* cause vascular Ehlers‐Danlos syndrome in one large Chinese family

**DOI:** 10.1111/jcmm.17063

**Published:** 2021-11-29

**Authors:** Mei Liang, Chong Chen, Yan Dai, Yunbing Chang, Yushun Gao

**Affiliations:** ^1^ Department of Thoracic Surgery National Cancer Center/National Clinical Research Center for Cancer/Cancer Hospital Chinese Academy of Medical Sciences and Peking Union Medical College Beijing China; ^2^ Department of Spine Surgery Guangdong Provincial People's Hospital Guangdong Academy of Medical Sciences Guangzhou China; ^3^ Department of Radiology First Affiliated Hospital Sun Yat‐Sen University Guangzhou China

**Keywords:** aneurysm, *COL3A1* gene, Ehlers‐Danlos syndrome vascular type, missense variant

## Abstract

Vascular Ehlers‐Danlos syndrome (vEDS) is a rare and severe hereditary connective tissue disease arising from a mutation in the type III collagen alpha I chain (*COL3A1*) gene, with a poor prognosis due to exceptional vascular ruptures and premature death. Herein, starting from a 36‐year‐old Chinese male patient with a complaint of upper abdominal pain, we collected clinical data of and performed a genetic analysis of a total of 20 family members. We identified two closely spaced *COL3A1* missense variants in *cis*, p.Leu734Phe (c.2199_2200TC>AT) and p.Gly741Ser (c.2221G>A), as the cause of vEDS in this family. p.Gly741Ser, a glycine substitution mutation, has been previously reported, whereas p.Leu734Phe, a non‐glycine substitution mutation, is novel. We analysed their independent and combined effects on the COL3A1 level in transfected skin fibroblast cells by means of Western blotting. We found that both variants independently led to a reduced COL3A1 level and, when combined, led to an even more reduced COL3A1 level compared to the wild type. Thus, each missense variant can be independently classified as a pathogenic variant, albeit with a synergetic effect when occurring together. Moreover, our genetic findings provide an explanation for four previous sudden deaths and identified two high‐risk carriers in the family.

## 1 INTRODUCTION

Vascular Ehlers‐Danlos syndrome (vEDS, also known as Ehlers‐Danlos type IV; OMIM# 130050) is a rare inherited autosomal dominant disorder (prevalence, 1/50,000–200,000) that is mainly characterized by fatal arterial and gastrointestinal complications.^1^, ^2^, ^3^, ^4^ vEDS is usually caused by heterozygous mutations in the *COL3A1* gene that codes for the pro‐alpha 1 chain of type III procollagen.^5^, ^6^, ^7^, ^8^, ^9^ The clinical diagnosis of vEDS is typically based on the standards laid down by an expert group in 1997.^2^ Thus, ecchymosis, thin skin with an evident venous pattern and typical facial characteristics lead to the diagnosis. Nonetheless, in most cases, the diagnosis is not suspected until the time an aneurysm and/or grooves in the arteries, crevices in the bowel or rupture of organs occurs. Significant phenotypic heterogeneity is also exhibited within family members in terms of disease onset and severity and affected organs. Additionally, the clinical features of vEDS may overlap with Loeys‐Dietz syndrome and Marfan syndrome, further complicating the clinical diagnosis.^3^, ^4^ Therefore, genetic tests are often required to establish a formal diagnosis.

Type III collagen is a critical structural protein in the walls of the vascular system and in the walls of hollow organs. Structural defects or lower levels of type III procollagen resulting from *COL3A1* mutations thus underlie the escalated ecchymosis, bowel and arterial frailty, and vaginal, uterine and cervical frailty in pregnancy as well as in delivery in vEDS patients. To date, a diverse range of loss‐of‐function variants in the *COL3A1* gene have been reported in the literature, with most of them being heterozygous glycine substitutions that occurred within the [Gly‐X‐Y]_343_ repeat of the type III procollagen.^6^ In this regard, Bowen and colleagues recently created two mouse models of vEDS, each carrying a heterozygous glycine substitution mutation (i.e., p. Gly209Ser or p. Gly938Asp) in *Col3a1*. They showed that Col3a1 structural deficiencies conferred by the glycine substitution mutations led to signalling abnormalities in the PLC/IP3/PKC/ERK (phospholipase C/inositol 1,4,5‐triphosphate/protein kinase C/extracellular signal‐regulated kinase) pathway that in turn mediated the risk of vascular rupture.^10^ Apart from these typical glycine substitution missense mutations, atypical non‐glycine substitution missense mutations (e.g., glutamic acid to lysine (Glu>Lys) substitutions) in the *COL3A1* gene have also been increasingly recognized to cause the disease.^6^, ^11^

In the present study, we describe the identification and functional analysis of two closely spaced *COL3A1* missense variants in *cis*, one being a typical glycine substitution missense mutation and the other being an atypical non‐glycine substitution missense mutation, as the cause of vEDS in a large Chinese family that affected more than 10 members across four generations. We show that the two missense variants had a synergetic effect on reducing the level of COL3A1 in transfected cells, providing novel insights into the pathogenesis of vEDS.

## 2 MATERIALS AND METHODS

### 2.1 Ethics statement

Informed consent was obtained from all participants (or parents/guardians when the participants were under the age of 18). The study protocol conformed to the ethical guidelines of the 1975 Declaration of Helsinki and was approved by the human research committee of Cancer Hospital, Chinese Academy of Medical Sciences and Peking Union Medical College and Guangdong Provincial People's Hospital, Guangdong Academy of Medical Sciences.

### 2.2 Proband and family members

A 36‐year‐old Chinese male patient (III:9; Figure 1) was hospitalized at Cancer Hospital, Chinese Academy of Medical Sciences and Peking Union Medical College, Beijing, China, with a complaint of upper abdominal pain. On physical examination, he had velvety, smooth, thin skin with visible veins in the hands and feet, acrogeria of the limbs and ecchymosis **(**Figure 2A). Moreover, vascular abnormalities were found by means of computed tomography angiography (CTA) (Figure 3A, B).

**FIGURE 1:**
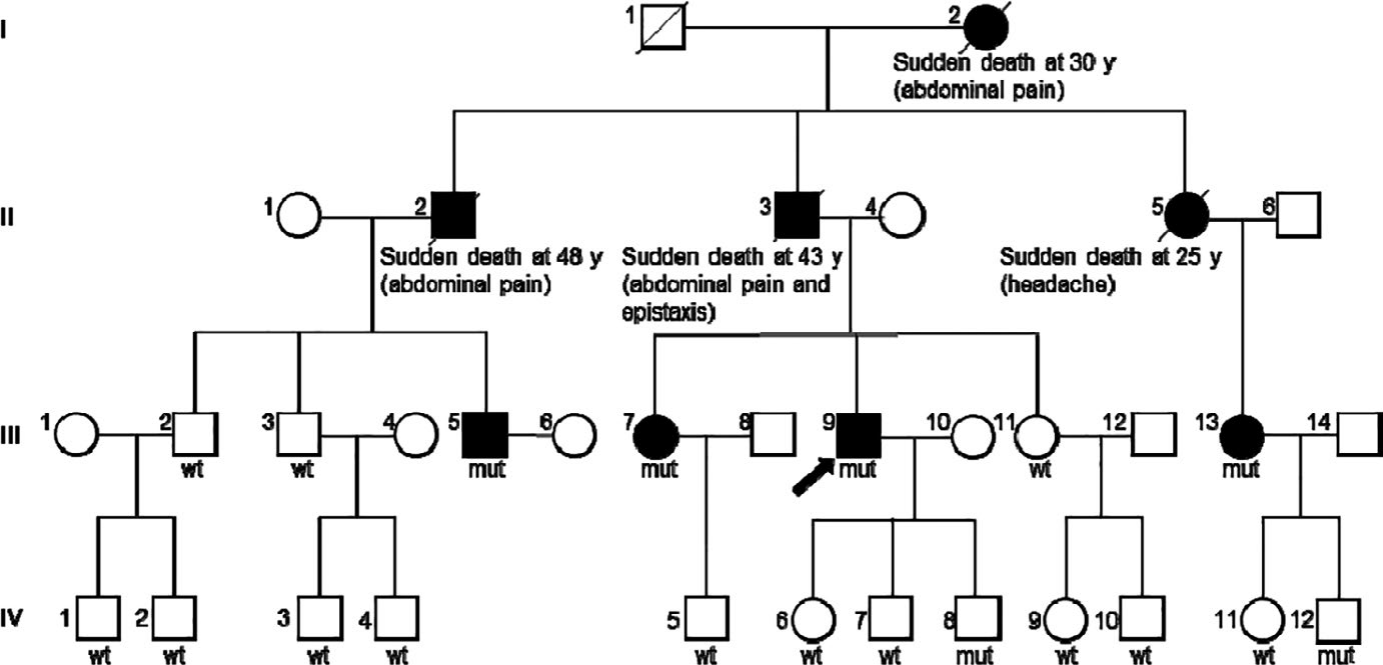
Pedigree of the studied Chinese family with vascular Ehlers‐Danlos syndrome. Filled symbols indicate clinically affected individuals, while open symbols indicate clinically unaffected family members. Arrow indicates the proband. Presence or absence of the two closely spaced *COL3A1* missense variants in *cis*, p.[Leu734Phe; Gly741Ser] (c.[2199_2200TC>AT; 2221G>A]) in the genetically analysed subjects is indicated by wt (wild type) or mut (mutation)

**FIGURE 2:**
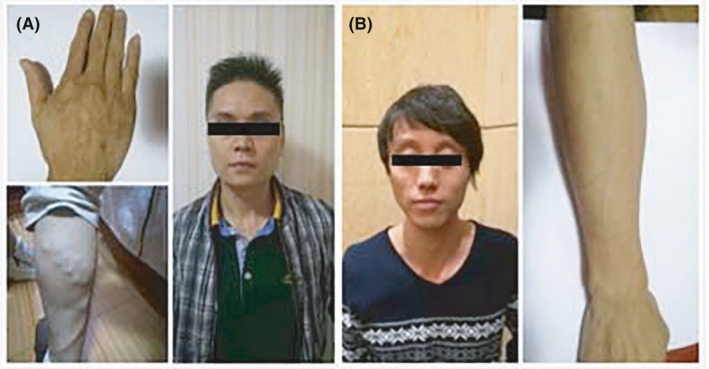
Clinical features of two patients under study. (A) The proband (III:9; see Figure 1) had acrogeria, thin skin, varicose veins and characteristic facial features. (B) Patient III:5 (see Figure 1) had acrogeria, thin skin and characteristic facial features. Consent for publication of the photographs was obtained from the patients

**FIGURE 3:**
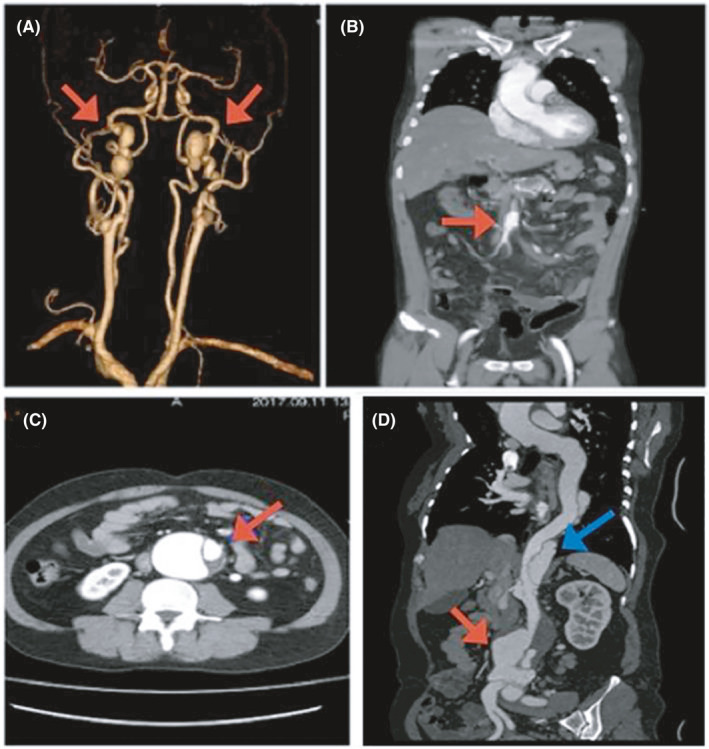
Vascular abnormalities in two patients with vascular Ehlers‐Danlos syndrome. (A) Coronal colour‐coded 3D volume‐rendered image showing bilateral internal carotid artery aneurysms (arrows) in the proband (III:9; see Figure 1). (B) Coronal contrast‐enhanced computed tomography angiography (CTA) showing a superior mesenteric artery dissection aneurysm (arrow) in the proband. (C) Axial contrast‐enhanced CTA showing type B aortic dissection (arrow) in the proband's 39‐year‐old elder sister (III:7; see Figure 1). (D) Coronal contrast‐enhanced CTA showing type B aortic dissection (blue arrow) and abdominal aortic aneurysm (red arrow) in III:7

Questioning about family history revealed that his grandmother (I:2), father (II:3) and two uncles (II:2 and II:5**)** (Figure 1) died suddenly of unexplained abdominal pain, headache or epistaxis when they were 25–48 years of age.

All living blood relatives of the proband were clinically assessed at the Cancer Hospital, Chinese Academy of Medical Sciences and Peking Union Medical College, Guangdong Provincial People's Hospital and First Affiliated Hospital of Sun Yat‐Sen University. The proband's 31‐year‐old cousin (III:5; Figure 1) had hyperextensible skin, ecchymosis, scarring and joint hypermobility. His 39‐year‐old elder sister (III:7; Figure 1) was diagnosed with Stanford type B aortic dissection and abdominal aortic aneurysm by means of CTA (Figure 3C, D) without any symptoms. His 41‐year‐old female cousin (III:13; Figure 1) had intracranial arterial rupture. The other blood relatives were healthy (Table 1).

**TABLE 1:** Clinical and genetic findings in a large Chinese family with vascular Ehlers‐Danlos syndrome

Family members	Age^a^	Age at first complication or diagnosis	Sex	Thin skin	Complication			CTA finding^b^	Sudden deathdeath		Variants of *COL3A1*
					Bowel rupture	Arterial dissection or rupture	Pneumothorax		Age	Symptom	
I:2	Deceased	‐	F	‐	‐	‐	‐	‐	30	Abdominal pain	‐
II:2	Deceased	‐	M	‐	‐	‐	‐	‐	48	Abdominal pain	‐
II:3	Deceased	‐	M	‐	‐	‐	‐	‐	43	Abdominal pain, epistaxis	‐
II:5	Deceased	‐	F	‐	‐	‐	‐	‐	25	Headache	‐
III:2	37	No	M	No	No	No	No	No	No	No	Negative
III:3	33	No	M	No	No	No	No	No	No	No	Negative
III:5	31	31	M	Yes	No	No	No	No	No	No	Positive
III:7	39	39	F	No	No	Yes	No	Yes	No	No	Positive
**III:9 (proband)** III 9	36	36	M	Yes	Yes	Yes	No	Yes	No	No	Positive
III:11	31	No	F	No	No	No	No	No	No	No	Negative
III:13	41	No	F	No	No	Yes	No	Yes	No	No	Positive
IV:1	6	No	M	No	No	No	No	No	No	No	Negative
IV:2	14	No	M	No	No	No	No	No	No	No	Negative
IV:3	13	No	F	No	No	No	No	No	No	No	Negative
IV:4	7	No	M	No	No	No	No	No	No	No	Negative
IV:5	19	No	M	No	No	No	No	No	No	No	Negative
IV:6	10	No	F	No	No	No	No	No	No	No	Negative
IV:7	13	No	M	No	No	No	No	No	No	No	Negative
IV:8	0.7	No	M	No	No	No	No	No	No	No	Positive
IV:9	8	No	F	No	No	No	No	No	No	No	Negative
IV:10	6	No	M	No	No	No	No	No	No	No	Negative
IV:11	18	No	F	No	No	No	No	No	No	No	Negative
IV:12	16	No	M	No	No	No	No	No	No	No	Positive

Abbreviation: CTA, computed tomography angiography.

^a^ In the year when the proband was hospitalized.

^b^ III:7, type B abdominal aortic dissection aneurysm; III:9, superior mesenteric artery dissection; III:13, intracranial arterial rupture.

### 2.3 Chip capture high‐throughput sequencing

Chip capture high‐throughput sequencing for an eight‐gene panel (i.e., *ADAMTS2*, *B4GALT7*, *COL5A1*, *COL5A2*, *PLOD1*, *COL3A1*, *SLC39A13* and *COL1A1*) was performed on the proband. In brief, genomic DNA was isolated, and the library was prepared; target gene‐coding regions were captured and enriched; and high‐throughput sequencing was conducted for subsequent mutation identification. The total length of the target regions was 26,733 bp, with an average sequencing coverage of 99.87% and an average sequencing depth of 152.15. Two rare missense variants in the *COL3A1* gene were identified in the proband.

### 2.4 Genotyping of the two *COL3A1* missense variants

The two *COL3A1* missense variants were analysed in all blood relatives of the proband by means of Sanger sequencing. The forward and reverse primers used for PCR amplification were 5′‐GAACGTGGACCTCCTGGAT‐3′ and 5′‐TGAAAATCAGCCAAGAAGAGG‐3′ respectively.

### 2.5 Plasmid constructs

The full‐length wild‐type human *COL3A1* cDNA (amplified using forward primer 5′‐ GAACGTGGACCTCCTGGAT‐3′ and reverse primer 5′‐TGAAAATCAGCCAAGAAGAGG‐3′) was cloned into the AgeI‐digested GV287 (Ubi‐MCS‐3FLAG‐SV40‐EGFP) vector (GeneChem Incorporation). This wild‐type *COL3A1* expression vector was then used to generate three variant expression vectors that carried the two *COL3A1* missense variants either separately or in combination by means of the QuikChange Lightning Site‐Directed Mutagenesis kit (Stratagene).^12^ The accuracy of the introduced variants was verified by Sanger sequencing.

### 2.6 Transfection and Western blotting

Transfection of skin fibroblasts with wild‐type and variant *COL3A1* plasmids was accomplished using Lipofectamine 3000 Transfection Reagent (Life Technologies) in accordance with the manufacturer's instructions. We isolated cytoplasmic proteins from the transfected cells using cytoplasmic extraction reagents (Thermo Fisher Scientific). Using a previously established protocol, we conducted a Western blotting assay with the following primary antibodies: anti‐Flag (Sigma) and anti‐GAPDH (Cell Signaling Technology) antibodies.^13^

### 2.7 Semiquantification of blot findings and statistical analysis

All computations were performed using the Image Processing and Analysis program in Java (ImageJ). We employed one‐way analysis of variance to statistically analyse the values. Kruskal‐Wallis tests were performed at significance levels of 5% and 1% using GraphPad Prism 5.^14^

### 2.8 Reference mRNA sequences and variant nomenclature

NM_000090.4 was used as the reference *COL3A1* mRNA sequence. Variants were named in accordance with Human Genome Variation Society recommendations (http://varnomen.hgvs.org/).

## 3 RESULTS

### 3.1 Genetic findings

**FIGURE 4:**
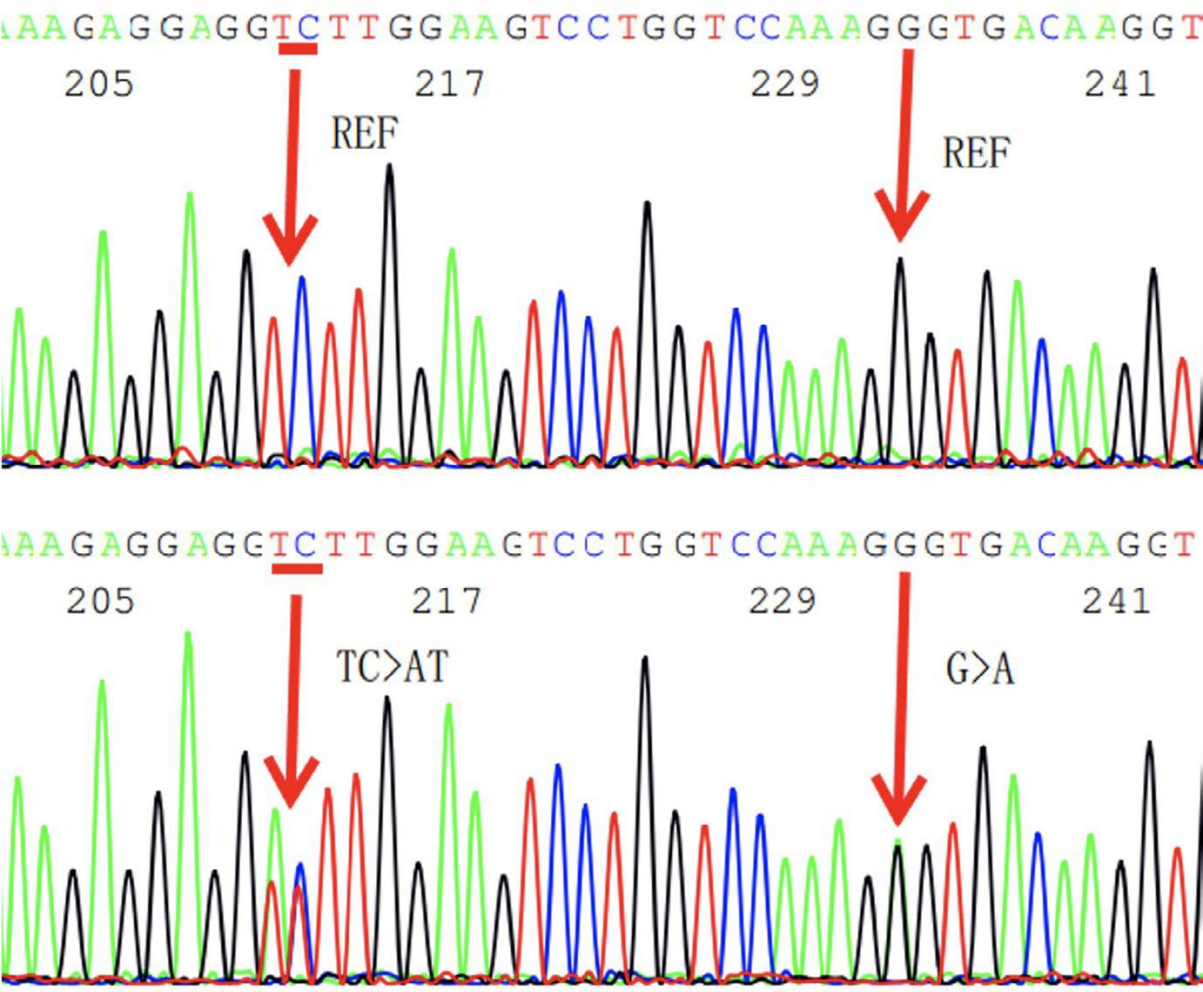
Sanger sequencing electropherogram showing the two heterozygous *COL3A1* missense variants identified in the proband and multiple family members. Upper panel: negative control; lower panel, proband. The two missense variants were determined to be in *cis* by virtue of their segregation pattern in the family and thus named p.[Leu734Phe; Gly741Ser] (c.[2199_2200TC>AT;2221G>A])

Genetic exploration performed on the proband (III:9; Figure 1) using next‐generation sequencing identified two missense variants in the *COL3A1* gene. The first was p. Leu734Phe (c.2199_2200delinsAT), which has not been previously reported in the literature. The second was p. Gly741Ser (c.2221G>A), a known pathogenic mutation.^6^ The two variants were confirmed by Sanger sequencing (Figure 4). Both variants are absent in gnomAD (https://gnomad.broadinstitute.org/; as of 25 October 2021).

We analysed the two missense variants by means of Sanger sequencing in all living blood relatives of the proband. Of the 20 individuals analysed (including the proband), the two variants were present together in six subjects and absent together in the remaining 14 subjects (Figure 1). This demonstrates that the two variants are located on the same chromosome. Since they are separated by merely 20 bases, we describe them as two closely spaced missense *COL3A1* variants in *cis*. Their formal nomenclature in accordance with Human Genome Variation Society recommendations is c.[2199_2200TC>AT;2221G>A] (p.[Leu734Phe; Gly741Ser]).

Protein function prediction was performed using SIFT and PolyPhen software as previously described.^15^ Both variants were predicted to be harmful.

### 3.2 Functional characterization of the two missense variants in transfected skin fibroblasts

**FIGURE 5:**
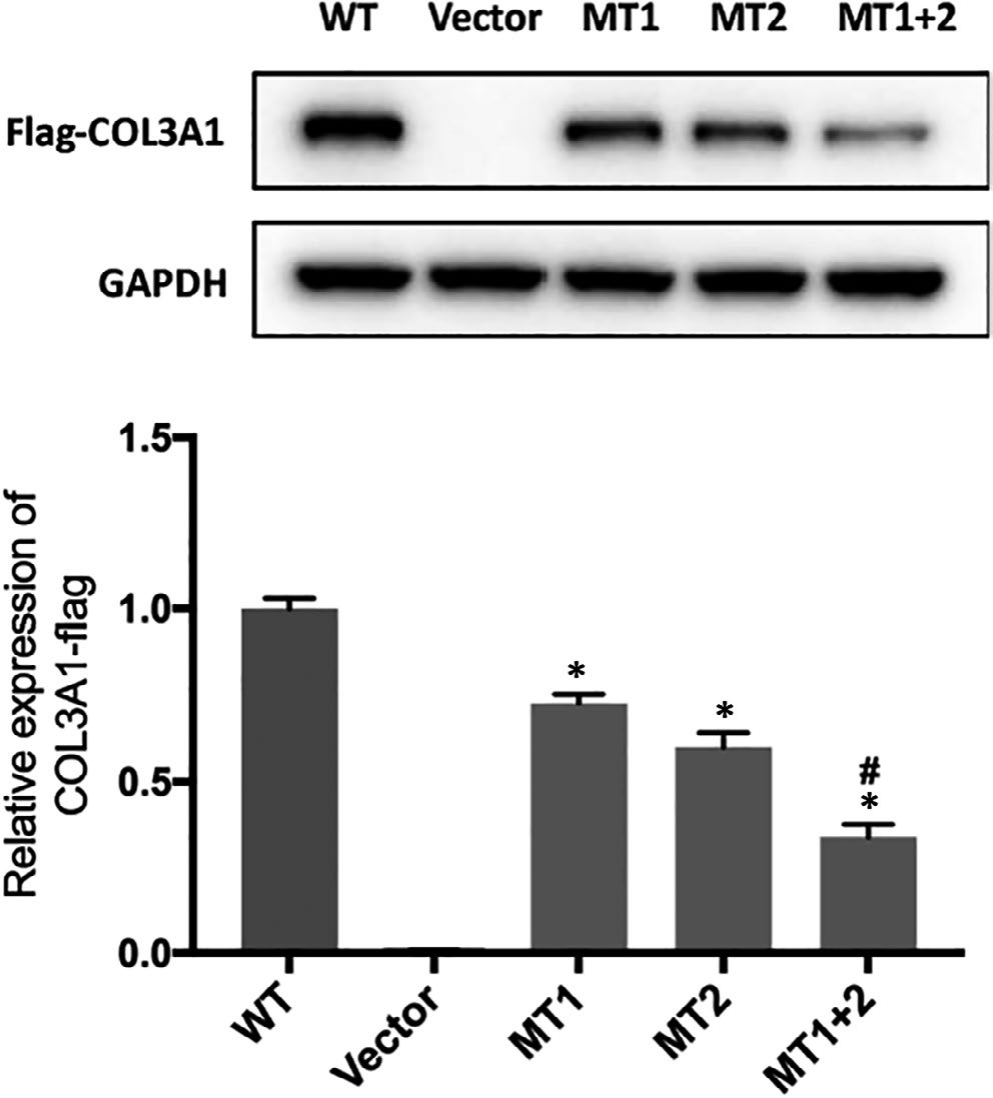
Western blot analysis of COL3A1 in transfected cells. WT, wild type; vector, empty vector; MT1, p. Gly741Ser alone; MT2, p. Leu734Php alone; MT1+2, the two missense variants in combination. GAPDH was used as the loading control

We employed a Western blot assay to study the expression of the wild‐type and variant COL3A1 proteins in transfected skin fibroblasts. Each variant in isolation led to a significantly reduced level of COL3A1, and the two variants in combination led to a more profound reduction in the level of COL3A1 in the transfected cells compared to the wild type **(**Figure 5).

## 4 DISCUSSION

Ehlers‐Danlos syndromes constitute a pathologically and genetically heterogeneous class of inheritable connective tissue defects, manifested by joint hypermobility, unusual skin elasticity and tissue fragility. The Villefranche nosology identifies six EDS subcategories, namely typical, hypermobile, vascular, kyphoscoliotic, arthrochalasic and dermatosparaxic EDS.^16^ vEDS is the most severe form of EDS; vEDS patients often suffer from sudden death due to large artery rupture. In mouse models, homozygous *Col3a1* mutations cause premature death mimicking that in humans. The cellular and biochemical impacts of *COL3A1* mutations have been the subject of extensive investigation. In particular, type III collagen with a glycine substitution mutation showed reduced thermal stability, thereby being more vulnerable to protein‐degrading enzymes. Mutant COL3A1 proteins were also prone to intracellular retention. Additionally, ultrastructural studies revealed dilatation of the rough endoplasmic reticulum and a shift in the diameter of the collagen fibres.^17^

In the present study, a large pedigree was built starting from the proband. The identification of two closely spaced *COL3A1* missense variants in *cis* in the proband and all affected family members firmly established the diagnosis of vEDS. This provides a retrospective explanation for the sudden deaths of the proband's grandmother, father and two uncles (Figure 1). Herein, it should be emphasized that we collected detailed clinical data of and genotyped the pathogenic *COL3A1* variants in all living blood relatives (*n* = 19) of the proband. To the best of our knowledge, such a large and well‐informed vEDS family has never been reported in the literature.

The identification of two closely spaced *COL3A1* missense variants in *cis*, p. Leu734Phe and p. Gly741Ser, as the genetic cause of vEDS in a large Chinese family, is of particular interest. p. Gly741Ser, a typical glycine substitution missense mutation, has previously been reported in vEDS patients.^6^ In contrast, p. Leu734Phe, a non‐glycine substitution missense mutation, is novel. Since the two variants are located on the same chromosome, we analysed their independent and combined effects on the COL3A1 level in transfected cells by means of Western blotting. We found that both variants independently led to a reduced COL3A1 level and, when combined, led to an even more reduced COL3A1 level. These findings suggest that each missense variant can be independently classified as a pathogenic variant, albeit with a synergetic effect when occurring together. We are not aware of any similar findings in the literature.

The two closely spaced *COL3A1* missense variants in *cis* were found not only in all clinically affected members but also in two healthy family members, IV8 and IV12 (Figure 1). It should, however, be pointed out that while IV8 was less than 1 year old, IV12 was 16 years old. In this regard, it is worth mentioning that in a European cohort study, only 17% of vEDS patients had experienced an initial complication by the age of 20.^6^ Thus, genetic counselling should be given to IV8 and IV12 about their risk of developing vEDS manifestations.

In summary, we have for the first time reported a combination of a typical glycine substitution missense mutation and an atypical non‐glycine substitution missense mutation as the cause of vEDS in an unusually large Chinese family. In fact, this is the first report of such a combination ever described to cause vEDS in the literature. More importantly, we demonstrated that both variants are independently functional and, when combined, confer a more severe effect on the structure/function of COL3A1.

## CONFLICT OF INTEREST

The authors confirm that there are no conflicts of interest.

## AUTHOR CONTRIBUTIONS

**Mei Liang:** Conceptualization (equal); Data curation (equal); Investigation (equal); Methodology (equal); Project administration (equal); Writing‐original draft (equal). **Chong Chen:** Conceptualization (equal); Data curation (equal); Investigation (equal); Methodology (equal); Project administration (equal); Writing‐original draft (equal). **Yan Dai:** Investigation (equal); Software (supporting). **Yunbing Chang:** Methodology (equal); Software (supporting); Supervision (supporting). **Yushun Gao:** Supervision (lead); Writing‐review & editing (lead).

## Data Availability

All data generated or analysed during this study are included in this article.
